# The Health-Related Quality of Life of German Desmoid Patients: Results from the PROSa-DES and PROSa Study

**DOI:** 10.3390/cancers18061046

**Published:** 2026-03-23

**Authors:** Martin Eichler, Rebekka Hoffmann, Christina Baumgarten, Jens Jakob, Bernd Kasper, Stephan Richter, Daniel Pink, Robert Grützmann, Jochen Schmitt, Markus K. Schuler, Peter Hohenberger

**Affiliations:** 1 National Center for Tumor Diseases (NCT/UCC) Dresden, Medical Faculty, Technical University Dresden, 01307 Dresden, Germany; 2 SOS-Desmoid, e.V., SPAEN Sarcoma Patients EuroNet e.V, 61200 Wölfersheim, Germany; 3 Sarcoma Unit, Mannheim Cancer Center, Mannheim University Medical Center, University of Heidelberg, 68167 Mannheim, Germanybernd.kasper@medma.uni-heidelberg.de (B.K.); 4 Clinic and Polyclinic for Internal Medicine I, University Hospital Carl Gustav Carus, 01307 Dresden, Germany; 5 Sarcoma Center Berlin-Brandenburg, Helios Hospital Bad Saarow, University Hospital Greifswald, 17475 Greifswald, Germany; daniel.pink@helios-gesundheit.de; 6 Sarcoma Center Berlin-Brandenburg, Helios Hospital Berlin-Buch, University Hospital Greifswald, 17475 Greifswald, Germany; 7 Department of Surgery, University Hospital, 91054 Erlangen, Germany; 8 Center for Evidence-Based Healthcare, University Hospital Carl Gustav Carus and Medical Faculty, Technical University Dresden, 01307 Dresden, Germany; 9 Division of Surgical Oncology & Thoracic Surgery, Medical Faculty Mannheim of University of Heidelberg, 68135 Mannheim, Germany

**Keywords:** desmoid tumors, health-related quality of life, EORTC QLQ C30, DTF QoL

## Abstract

Desmoid-type fibromatosis (DT) is a rare, locally aggressive soft tissue tumor with a frequently chronic course and substantial impact on health-related quality of life (HRQoL). While international studies have demonstrated considerable symptom burden and psychosocial impairment, data from Germany are lacking. This study aimed to assess HRQoL in German patients and to identify factors associated with HRQoL. In this cross-sectional analysis, adult patients with histologically confirmed DT completed the EORTC QLQ-C30 and the disease-specific Desmoid-Type Fibromatosis Quality of Life Questionnaire (DTF-QoL). HRQoL scores were compared with reference data from the German general population, German sarcoma patients, and international DT cohorts. Sociodemographic, disease-, tumor-, and treatment-related factors associated with HRQoL were examined using multivariate linear regression analyses. A total of 155 patients were included (69.7% female; mean age 45.0 years). Compared with the German general population, DT patients reported clinically relevant impairments in role, social, and emotional functioning and higher symptom burden, particularly pain, fatigue, and insomnia. Compared with sarcoma patients, DT patients showed better physical, role, and social functioning, while emotional functioning and symptom burden were largely comparable. German DT patients reported consistently worse HRQoL than Dutch/UK cohorts and moderately worse outcomes than Indian cohorts. Female gender, unemployment or disability pension, intensive multimodal treatment, multiple lines of systemic therapy, and tumor location in the lower extremities were independently associated with poorer HRQoL. DT is associated with sustained and clinically meaningful HRQoL impairment. HRQoL is driven primarily by psychosocial and treatment-related factors rather than disease duration, supporting the concept of DT as a chronic condition requiring long-term, multidisciplinary supportive care.

## Simple Summary

Desmoid-type fibromatosis is a rare soft tissue tumor that does not metastasize but can grow aggressively and cause substantial long-term symptoms. Many patients experience pain, fatigue, and psychological distress that affect daily life, work participation, and social relationships. However, data on the quality of life of desmoid patients in Germany are limited. This study evaluates health-related quality of life in a national cohort of German desmoid patients using both a general cancer quality-of-life questionnaire and a disease-specific instrument. The results show persistent impairments, particularly in emotional, social, and role functioning. Women, unemployed patients, and those who received intensive or multimodal treatments reported the greatest burden. These findings suggest that desmoid-type fibromatosis should be regarded as a chronic condition requiring long-term multidisciplinary care that integrates medical treatment with psychosocial and supportive interventions.

## 1. Introduction

Desmoid-type fibromatosis (DT) is a rare, locally aggressive, and non-metastasizing soft tissue tumor that predominantly affects young adults, especially women. In the ICD-10 coding system, DTs are classified as D48.1 (tumors of uncertain behavior). The disease is defined as a clonal fibroblastic proliferation that can arise in every part of the body, with a significant subgroup arising at the site of prior trauma [1].

Two subgroups of the disease should be recognized particularly with respect to different morbidity features and co-morbidities. Sporadic desmoids develop due to a point mutation in the *CTNNB1* gene coding for β-catenin [2,3], whereas patients with familial adenomatosis polyposis (FAP) suffer from FAP-associated Gardner syndrome. The latter group typically develops DTs at the mesentery after restorative colectomy and the mechanism is based on a mutation in the *APC* gene [4]. DT can cause considerable morbidity due to its infiltrative growth and high risk of local recurrence after surgery [5].

International studies have shown that DT patients experience a broad spectrum of symptoms and psychosocial challenges, including pain, fatigue, functional impairments, and fear of disease progression. These factors can substantially affect key domains of daily life, such as employment, family planning, and social participation [6,7]. However, systematic investigations of health-related quality of life (HRQoL) in DT remain scarce. The development of disease-specific instruments such as the Desmoid-Type Fibromatosis Quality of Life Questionnaire (DTF-QoL) [8] and the Global Desmoid Symptom Scale (GODDESS) [9,10] has enabled a more detailed assessment of symptom burden and functional limitations in this patient group.

Existing evidence indicates that DT patients report markedly lower HRQoL than the general population, with pronounced deficits in physical, role, and social functioning, and elevated levels of pain and fatigue [11,12]. Schut et al. (2022) demonstrated substantial symptom burden and reduced HRQoL among Dutch and United Kingdom (UK) DT patients [13], while Garg et al. (2022) reported similarly low HRQoL scores and high psychosocial distress in an Indian cohort of DT patients [14]. Although desmoid-type fibromatosis is histologically benign and does not metastasize, its clinical course often resembles that of chronic soft tissue sarcomas in terms of local aggressiveness, repeated treatments, and long-term functional impairment. Because sarcoma patients represent a well-characterized oncological population with established HRQoL reference data, comparison with sarcoma cohorts provides a clinically meaningful benchmark for contextualizing the burden experienced by desmoid patients.

To date, no corresponding data are available from Germany. The present study therefore provides the first comprehensive analysis of HRQoL among German patients with desmoid tumors by combining data from two nationwide cohort studies (PROSa-DES and PROSa). By integrating both the generic EORTC QLQ-C30 and the disease-specific DTF-QoL questionnaire, the study allows a detailed characterization of symptom burden and psychosocial impact and enables the identification of clinical and sociodemographic factors associated with HRQoL in this rare disease. Against this background, the present study had two objectives:

(a) to evaluate HRQoL in German DT patients in context by comparing their outcomes with those of the general population, sarcoma patients, and international DT cohorts, and
(b) to identify sociodemographic and clinical factors associated with variations in HRQoL.

## 2. Methods

### 2.1. Study Design and Cohorts

This analysis uses cross-sectional data from the PROSa-DES study (Burden of Disease and Living Situation in Desmoid Patients) and the PROSa study (Burden and Medical Care of Sarcoma in Germany: Nationwide Cohort Study Focusing on Modifiable Determinants of Patient-Reported Outcome Measures in Sarcoma Patients).

PROSa-DES and PROSa were questionnaire-based, observational studies. In PROSa-DES, patients were recruited between November 2023 and June 2024. Desmoid-type fibromatosis (DT) patients included within the PROSa study were recruited between September 2017 and November 2020. The two datasets were combined for the present analysis, as recruitment of DT patients within the original PROSa framework had been interrupted by the COVID-19 pandemic.

Eligible participants were adults (≥18 years) with histologically confirmed sporadic desmoid-type fibromatosis, sufficient cognitive and linguistic ability to provide written informed consent, and the capacity to complete German study questionnaires. Patients with familial adenomatous polyposis (FAP)-associated DT were excluded.

The PROSa-DES study was approved by the Ethics Committee of the Technical University Dresden (EK489112023) and registered at ClinicalTrials.gov (NCT06258421). The PROSa study was approved by the Ethics Committee of the Technical University Dresden (EK1790422017) and registered at ClinicalTrials.gov (NCT03521531).

### 2.2. Data Collection and Variables

PROSa-DES participants were recruited through two pathways. (a) Patients were identified from medical records at the University Hospital Mannheim. (b) Additionally, patients were recruited via convenience sampling, using public calls for participation disseminated via the SOS Desmoid patient organization (sos-desmoid.de) and the German Sarcoma Foundation (sarkome.de).

In the PROSa study, participants were approached at one of 39 referral centers across Germany during outpatient visits, and in some cases by phone or letter [15].

In PROSa-DES, questionnaires were distributed, completed at home, and returned by mail, whereas in PROSa they could be completed by mail or online. Clinical data were extracted from medical records for patients recruited through hospital sites. For participants enrolled via convenience sampling (PROSa-DES), relevant clinical information was obtained from recent physician letters submitted by the participants themselves or—after written consent—directly from the treating physicians.

### 2.3. Patient-Reported Outcomes

Health-related quality of life (HRQoL) was assessed using two validated instruments.

First, the European Organisation for Research and Treatment of Cancer Quality of Life Core Questionnaire (EORTC QLQ-C30) [16] was applied to measure global health status, functional domains (physical, role, emotional, cognitive, and social functioning), and symptom scales (e.g., pain, fatigue, nausea/vomiting).

Second, the Desmoid-Type Fibromatosis Quality of Life Questionnaire (DTF-QoL) [8] was used as a disease-specific measure, capturing desmoid-related symptoms across four symptom scales and the psychosocial impact across eleven impact scales.

The EORTC QLQ-C30 was selected because it represents the most widely used oncology-specific HRQoL instrument and allows comparison with large international cancer reference populations. In addition, the disease-specific DTF-QoL questionnaire was included to capture symptoms and psychosocial impacts specific to desmoid-type fibromatosis. The combination of a generic oncology-specific and a disease-specific instrument allows a comprehensive assessment of HRQoL in this patient population.

Both instruments were scored according to their respective scoring manuals. Higher scores in global health status and functioning domains indicate better HRQoL, whereas higher scores in symptom or impact scales reflect greater symptom burden or psychosocial impairment.

### 2.4. Model Variables

Sociodemographic and clinical characteristics included age at diagnosis (18–35/36–55/>55 years; PROSa, PROSa-DES), gender (male/female/diverse; PROSa, PROSa-DES), employment status (employed/unemployed/disability pension/retirement pension/other; PROSa, PROSa-DES), and educational level (low/medium/high; PROSa, PROSa-DES).

Disease- and treatment-related variables comprised time since diagnosis (0–<5/5–<10/>10 years; PROSa, PROSa-DES), time since treatment (in treatment/0–<5/5–<10/>10 years; PROSa-DES), number of systemic therapy lines (0–1 vs. ≥2; PROSa-DES), recurrence status (no/yes/no surgery; PROSa, PROSa-DES), received treatments (surgery only/systemic therapy only/chemotherapy + surgery/chemotherapy + surgery + radiotherapy/none [watch & wait]/other; simplified for regression: surgery ± systemic therapy/chemotherapy + surgery + radiotherapy/other; PROSa-DES), and tumor localization (lower extremity (incl. hip/pelvis)/upper extremity (incl. shoulder)/trunk/abdominal wall/intra-abdominal/head & neck/not defined or multifocal; PROSa, PROSa-DES).

### 2.5. Statistical Analysis

Descriptive statistics were calculated to characterize the study sample. Frequencies, proportions, means, and standard deviations (SD) were reported for all model variables. For each domain of the EORTC QLQ-C30 and the DTF-QoL, mean values and standard deviations were computed. Distribution of HRQoL scores was assessed using Shapiro–Wilk tests and graphical inspection (Supplementary Tables S1 and S2). Despite moderate deviations from normality in some scales, results are reported as means and standard deviations to ensure comparability with established EORTC reference data.

Mean scores were compared with reference data from four external sources:

(a) a German general population sample with reference data for the EORTC QLQ-C30 [17],
(b) a German sarcoma cohort from the PROSa study with EORTC QLQ-C30 data [18],
(c) a Dutch/UK desmoid tumor cohort with available data for both the DTF-QoL and the EORTC QLQ-C30 [13], and
(d) an Indian desmoid tumor cohort with EORTC QLQ-C30 data [14].

For visualization, radar plots were used to display functional and symptom scales (C30), while bar charts with error bars (mean ± SD) depicted DTF-QoL domains.

Univariate analyses and multivariable regression analyses were conducted for all 15 QLQ-C30 and 14 DTF-QoL scales. Linear regression was chosen because all HRQoL outcomes represent continuous scale scores ranging from 0 to 100. Regression coefficients (B) therefore represent the mean difference in HRQoL score associated with the respective predictor variable. The analysis of DTF-QoL scales was restricted to the PROSa-DES population. For univariate analyses, associations between each HRQoL scale and independent variables (as defined above) were examined using *t*-tests or ANOVA for categorical predictors. Results of univariate analyses are presented in the Supplementary. For multivariate analyses, variables with *p* < 0.10 in univariate testing were entered into generalized linear regression models (GENLIN procedure in SPSS). A backward selection procedure based on Wald statistics was applied, sequentially removing variables with *p* ≥ 0.20 until only variables below this threshold remained in the final model. All analyses were performed using SPSS Statistics V.30 (IBM, Armonk, NY, USA). Overall model fit was assessed using likelihood ratio chi-square statistics (Supplementary Table S3). Due to the exploratory nature of the analysis no corrections for multiple testing were applied. As this study represents an exploratory cross-sectional analysis based on existing observational cohort data from the PROSa-DES and PROSa studies, no formal a priori sample size calculation was performed. A two-sided *p*-value < 0.05 was considered statistically significant.

## 3. Results

### 3.1. Patient Characteristics

A total of 155 participants were included in the combined analysis, comprising 109 patients from the PROSa-DES cohort (70.3%) and 46 patients from the PROSa cohort (29.7%).

The majority of participants were female (69.7%), with comparable proportions across cohorts (PROSa-DES 69.7%; PROSa 69.6%). The mean age of the total sample was 45.0 years (SD = 14.3), with 45.7 years (SD = 14.1) in PROSa-DES and 43.4 years (SD = 14.8) in PROSa.

Regarding disease duration, 41.6% of all participants had been diagnosed within the past 5 years (PROSa-DES 26.6%, PROSa 77.8%), 28.4% between 5 and 10 years, and 30.0% more than 10 years prior to the survey. The mean time since diagnosis was 7.9 years (SD = 6.4) overall, 9.5 years (SD = 6.1) in PROSa-DES, and 4.0 years (SD = 5.4) in PROSa.

Nearly two thirds of all participants had completed secondary education (62.3%), and 73.5% were employed at the time of survey (62.4%). The most frequent tumor locations were the lower extremities (31.0%) and the intra-abdominal region (23.2%). Further sociodemographic and clinical variables are provided in Table 1.

### 3.2. Quality of Life of Desmoid Patients in Context

**Figure 1:**
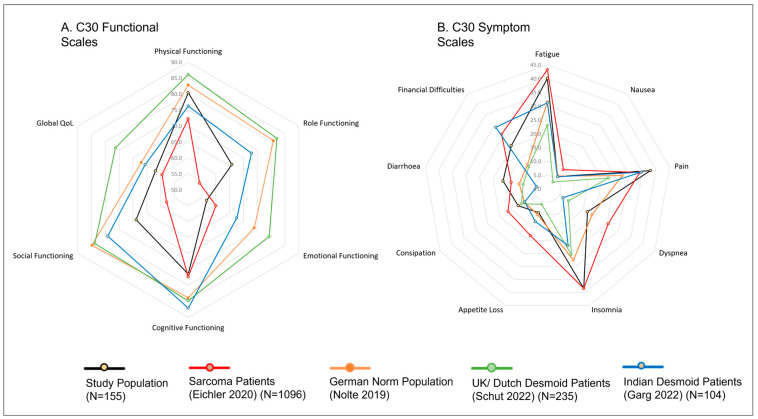
Comparison of EORTC-QLC-C30 Scales of the PROSa-DES Population with German Sarcoma Patients, German Norm Population, UK/Dutch Desmoid Patients and Indian Desmoid Patients.

**Figure 2:**
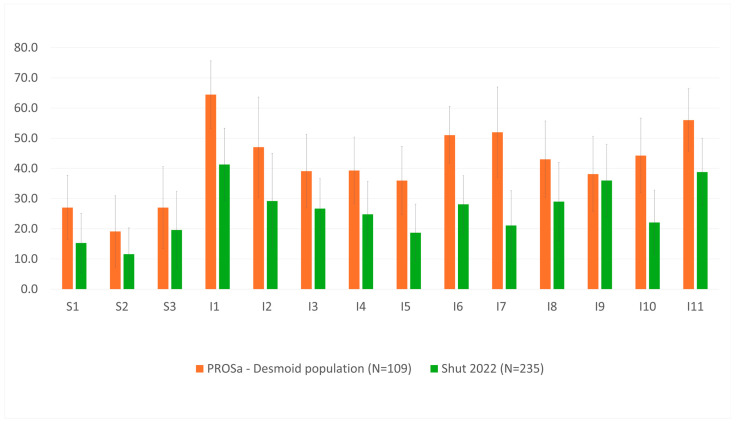
Comparison of DTF QoL Scales of the PROSa-DES Population with UK/Dutch Desmoid Patients (Shut 2022). Mean and Standard Deviation. S1 = Emotional and psychological consequences. S2 = Physical consequences. S3 = Pain and discomfort. I1 = Concerns about condition. I2 = Job and education. I3 = Doctor–patient relationship, communication and information. I4 = Effect of DT on relationships. I5 = Physical limitations and consequences. I6 = Diagnostic and treatment trajectory of DT. I7 = Parenting and fertility. I8 = Body image and sensation. I9 = Supportive care. I10 = Concerns around treatment and its consequences. I11 = Unpredictable course and nature of DT. DT = desmoid tumor.

To contextualize the HRQoL results, mean scores of the EORTC QLQ-C30 were compared with four reference populations: the German general population [17], German sarcoma patients [19], a Dutch/UK DT cohort [13] and an Indian DT cohort [14] (Figure 1). Mean scores of the DTF-QoL scores were compared with the Dutch/UK DT cohort [13] (Figure 2). Numeric results are presented in Supplementary Tables S4 and S5.

Compared to the German general population, global health status of DT patients was approximately 5 points lower. The largest differences in functional domains were observed in role, social, and emotional functioning (15–20 points below population norms). In the symptom scales, the most pronounced deviations were seen for pain, insomnia, and financial difficulties, with scores 10–15 points higher than population averages.

When compared with the German sarcoma cohort, desmoid patients reported slightly better global health (about 5 points higher), as well as higher physical, role, and social functioning (by 8–12 points). They were also less affected by dyspnea and appetite loss (≈9 points difference). Symptom burdens for fatigue, pain, and insomnia were largely comparable (differences ≤ 4 points).

Relative to the Dutch/UK desmoid cohort, German patients reported consistently higher burden and lower HRQoL, with differences of 15–23 points in role, social, and emotional functioning as well as global health, and of 10–17 points in fatigue, pain, insomnia, and financial difficulties.

Compared with the Indian desmoid cohort, German patients also showed higher impairments, although differences were less pronounced. HRQoL was particularly reduced in role, social, cognitive, and emotional functioning (7–11 points difference). Regarding symptoms, German patients reported markedly higher insomnia (17 points), and higher fatigue and dyspnea (9–10 points). In contrast, Indian patients experienced greater financial difficulties (9 points higher).

On the disease-specific level, DTF-QoL scores of German patients were higher across all domains, indicating greater burden. The most pronounced differences (≥20 points) were found for parenting and fertility (31 points), concerns about condition (23 points), and diagnostic and treatment trajectory (23 points).

### 3.3. Factors Associated with HRQoL

Results of the univariate analyses for all model variables and HRQoL domains (EORTC QLQ-C30 and DTF-QoL) are presented in the Supplementary Tables S4 and S5. Factors with *p* < 0.1 in univariate testing were included in the generalized linear regression models. Multivariate results are summarized in Table 2 (DTF-QoL) and Table 3 (EORTC QLQ-C30).

### 3.4. Sociodemographic Factors

Gender. Male participants reported a consistently lower burden and better HRQoL than female participants across almost all DTF-QoL domains. The largest differences were observed in parenting and fertility (B = −26.6, *p* < 0.001), effect on relationships (B = −19.2, *p* < 0.001), doctor–patient relationship (B = −17.6, *p* < 0.001), supportive care (B = −16.3, *p* < 0.001), and pain/discomfort (B = −15.7, *p* < 0.001). Similarly, on the EORTC QLQ-C30, men reported significantly better physical functioning (B = 13.1, *p* < 0.001) and emotional functioning (B = 16.2, *p* < 0.001), experienced less pain (B = −23.2, *p* < 0.001), and indicated a higher global HRQoL (B = 12.4, *p* < 0.01).

Age. Younger participants (< 35 years) showed higher burden in nausea/vomiting (B = −6.1, *p* = 0.01 for 36–55 years; B = −12.6, *p* < 0.001 for > 55 years) and parenting and fertility (B = −43.2, *p* < 0.001 for 36–55 years).

Education. No significant associations were observed between educational level and HRQoL in either the EORTC QLQ-C30 or the DTF-QoL domains.

Employment status. Involuntarily unemployed participants, including those receiving a disability pension, reported a substantially higher burden across multiple DTF domains compared to employed individuals. The most prominent differences were found in job and education (B = 44.5, *p* < 0.001), emotional consequences (B = 26.7, *p* < 0.001), physical consequences (B = 26.0, *p* < 0.001), and effect on relationships (B = 25.4, *p* < 0.001).

Consistent with these findings, unemployed participants also demonstrated significantly lower HRQoL in the EORTC QLQ-C30, with reductions across 13 of 15 domains. Marked differences were observed in physical (B = −27.4, *p* < 0.001), role (B = −33.0, *p* < 0.001), emotional (B = −23.8, *p* < 0.001), social functioning (B = −40.3, *p* < 0.001), and global HRQoL (B = −25.3, *p* < 0.001). In addition, they reported significantly higher symptom burden, particularly for fatigue (B = 31.8, *p* < 0.001), insomnia (B = 39.6, *p* < 0.001), and financial difficulties (B = 48.5, *p* < 0.001).

### 3.5. Factors Related to the Course of Disease

In the DTF-QoL, time since treatment showed no significant associations with any of the assessed domains. For time since diagnosis, higher burden in physical consequences was observed 5–10 years after diagnosis compared with patients diagnosed within the past 0–5 years (B = 11.5, *p* = 0.03).

In the EORTC QLQ-C30, participants with more than 10 years since treatment reported better cognitive functioning (B = 20.0, *p* = 0.02) and lower fatigue (B = −17.9, *p* = 0.04). Similarly, insomnia was less pronounced among participants diagnosed more than 10 years prior (B = −12.2, *p* = 0.03) compared with those diagnosed within the past 0–5 years.

Patients with a tumor recurrence reported lower burden in supportive care (B = −11.4, *p* = 0.03) compared with those without recurrence.

### 3.6. Treatment Related Factors

Patients who had received surgery, systemic therapy, and radiotherapy reported significantly higher burden in several DTF-QoL domains compared to those treated with surgery alone and/or systemic therapy. The most affected domains were physical consequences (B = 11.9, *p* = 0.02), physical limitations (B = 11.8, *p* = 0.02), and treatment-related concerns (B = 11.5, *p* = 0.03).

In the EORTC QLQ-C30, this group also demonstrated lower physical functioning (B = −15.3, *p* < 0.001), lower global HRQoL (B = −9.6, *p* = 0.02), and higher pain scores (B = 13.9, *p* = 0.04).

Similarly, participants who had undergone two or more lines of systemic therapy reported greater pain/discomfort (B = 19.0, *p* < 0.01), higher impact on body image (B = 15.3, *p* < 0.01), and more pronounced diarrhea (B = 13.4, *p* = 0.04) than those who had received one or no prior line of systemic therapy.

### 3.7. Tumor-Related Factors

Patients with tumors of the lower extremities reported the highest burden across several HRQoL domains. Compared with this reference group, participants with tumors located in the upper extremities (B = −17.2, *p* = 0.02), trunk (B = −13.0, *p* = 0.02), abdominal wall (B = −16.0, *p* < 0.01), and head and neck region (B = −18.2, *p* = 0.02) showed significantly lower physical consequences in the DTF-QoL. The domain parenting and fertility was less affected in patients with upper extremity tumors (B = −46.5, *p* < 0.01). Lower treatment-related concerns were also observed in tumors of the upper extremities (B = −24.3, *p* < 0.01), abdominal wall (B = −14.1, *p* = 0.02), and head and neck (B = −26.6, *p* < 0.01).

In the EORTC QLQ-C30, participants with tumors in the trunk (B = 12.8, *p* = 0.01) and abdominal wall (B = 11.4, *p* = 0.03) reported better physical functioning compared to those with tumors in the lower extremities.

## 4. Discussion

This study provides the first comprehensive assessment of health-related quality of life (HRQoL) in German patients with sporadic desmoid-type fibromatosis using both the disease-specific DTF-QoL and the generic EORTC QLQ-C30. By combining data from the PROSa-DES and PROSa cohorts, it offers a contextualized view of symptom burden, functioning, and psychosocial impact in a national sample of DT patients.

### 4.1. Quality of Life in Context

Compared with the German general population, DT patients showed substantial and clinically relevant impairments, particularly in role, social, and emotional functioning, as well as higher levels of pain, fatigue, and insomnia. The observed mean differences correspond to moderate to large effect sizes, indicating significant decrements in patient-perceived health status [20].

When compared with sarcoma patients, German DT patients reported higher scores in physical, role, and social functioning with differences exceeding the threshold for clinical relevance on the EORTC QLQ-C30 scales [20]. In contrast, emotional functioning and global health status were comparable between groups, suggesting that emotional well-being remains similarly affected in both DTF and malignant sarcoma populations. Likewise, pain, fatigue, and insomnia scores were largely comparable, indicating that symptom burden can reach malignant-like levels despite the benign histology of DT. Taken together, these findings illustrate that while DT patients experience better role and social participation than sarcoma patients, their emotional as well as aspects of their symptomatic burden remains clinically significant and largely unmitigated by the benign nature of the disease. Consistent with previous reports, chronic pain and fatigue are among the most frequent and distressing symptoms in DT, often persisting long after active treatment and substantially affecting sleep and daily functioning [13,14,21,22,23,24].

Notably, when compared with Dutch/UK DT cohorts, German patients showed large and clinically meaningful differences in several EORTC QLQ-C30 domains—particularly role, emotional, and social functioning—as well as a higher burden of fatigue, pain, and insomnia. Similarly, scores on the DTF-QoL were consistently worse in the German cohort, most prominently in the domains parenting and fertility, concerns about condition, and diagnostic and treatment trajectory. Comparisons with the Indian DT cohort revealed the same direction of effects, though differences were generally less pronounced.

A comparable pattern can be seen in European normative data reported by Nolte et al. [17], where the German general population scored lower in global health and emotional functioning than respondents from The Netherlands. When comparing the PROSa results with those of the Dutch SURVSARC study [25], German patients showed lower physical, role, and social functioning and higher levels of fatigue and pain. Beyond differences in healthcare organization, these cross-national variations may also reflect cultural differences in health perception and response behavior [6].

Additionally, the treatment history differed substantially between the Dutch/UK cohort and the German patients. In the Dutch/UK cohort, over one third of patients were managed with active surveillance only, compared with less than 10% in the present cohort. The proportion of patients receiving drug therapy was comparable, whereas surgery had been performed far more frequently in the German cohort (67% vs. 27%) [13]. This more intensive treatment history likely reflects longer disease duration and surgical management during periods when active surveillance was not yet established as a reasonable initial strategy. Consequently, patients may have been exposed to more extensive information about potential complications and side effects, which could contribute to heightened concern and distress compared with an approach that begins with observation and reserves treatment for disease progression.

The observed disparities are therefore likely multifactorial, shaped by differences in healthcare structures, treatment strategies, social support systems, and cultural interpretations of quality-of-life constructs, rather than being solely attributable to disease-specific mechanisms. To contextualize these differences, comparisons with international cohorts are important. The Indian cohort was included as an additional reference because it represents one of the few published datasets reporting EORTC QLQ-C30 outcomes specifically for desmoid tumor patients, although cross-national comparisons inevitably reflect differences in healthcare systems and cultural perceptions of health.

### 4.2. Determinants of HRQoL

#### 4.2.1. Gender

Gender consistently emerged as a key determinant of HRQoL in desmoid-type fibromatosis. Across both generic and disease-specific measures, women reported lower functioning and higher symptom burden than men, confirming findings from previous DT [13,22,26], and broader oncology research [27]. These differences are probably attributable to a complex interplay of biological, psychological, and sociocultural factors—such as hormonal influences, greater emotional expressiveness, higher body-image sensitivity, and differing coping styles. The patterns suggest that female patients represent a particularly vulnerable group in whom targeted psycho-oncological and social support interventions may be especially beneficial.

#### 4.2.2. Employment Status

Employment status was another strong and consistent predictor of HRQoL. Patients who were unemployed or receiving disability pensions reported substantially poorer functioning and higher symptom burden than those in active employment. This observation aligns with previous evidence from the PROSa study [28]. Similar patterns have been observed elsewhere, where work participation is closely associated with improved HRQoL outcomes [29,30]. Beyond better financial stability, employment provides structure and can create a sense of purpose, all of which contribute to psychological well-being and resilience. Loss of work or occupational disability may intensify fatigue, anxiety, and social withdrawal. These findings highlight the importance of early vocational counseling and workplace reintegration programs as key components of care for DT patients.

### 4.3. Disease-Related Factors

The duration since diagnosis or treatment showed only limited associations with HRQoL, suggesting that neither time since treatment nor time since diagnosis are always reliable predictors of recovery. Improvements were reported in fatigue, cognitive functioning, and insomnia, while HRQoL in most other domains remained stable over time rather than showing continuous improvement. In contrast, the highest burden of physical consequences was reported 5–10 years after diagnosis, indicating a prolonged impact of the disease on daily life. These findings are consistent with previous research in DT [13], which similarly observed persistent functional and psychosocial burden even many years after diagnosis. The observed inconsistent time effects indicate that DT behaves as a chronic condition with enduring physical and emotional consequences, rather than a disease from which patients fully recover once treatment ends. This chronicity may reflect both the hardly predictable clinical course—with repeated recurrences and prolonged surveillance—and sustained psychological processes such as fear of progression or adaptation fatigue. Consequently, long-term care for DT patients should not rely solely on time as a healing factor but include ongoing monitoring, symptom management, and psychosocial support to address persistent challenges in daily life.

### 4.4. Treatment-Related Factors

Treatment modality and intensity emerged as important determinants of HRQoL. Patients who had received combined local and systemic therapies—particularly multimodal regimens involving surgery, radiotherapy, and systemic treatment—reported lower physical functioning and global QoL, together with higher levels of pain and treatment-related concerns. These results mirror findings from previous DT studies showing that treatment intensity and invasiveness are among the strongest contributors to long-term symptom burden [13]. Repeated or multimodal interventions may amplify pain, fatigue, and fear of recurrence, while also affecting body image and social participation.

Similarly, patients who underwent multiple lines of systemic therapy experienced greater discomfort and poorer body image, consistent with cumulative treatment-related toxicity and the psychological toll of repeated therapeutic failure [31]. Such associations emphasize that even in a disease managed with curative intent, the cost of achieving local control can be substantial in terms of long-term quality of life. Despite worldwide consensus and prospective randomized trials [6,24,32,33] there was no registered drug available until very recently (Nirogacestat, and Sorafenib in Germany since August 2024 by GBA allowance). This might also contribute to the level of anxiety about whether access to therapy will be available once required by disease progression

### 4.5. Tumor Location

Tumor site was another relevant determinant of HRQoL. Patients with tumors of the lower extremities reported the highest functional limitations and physical consequences, whereas those with lesions of the trunk, abdominal wall, or head and neck experienced comparatively better functioning and less treatment-related concern. Similar patterns have been observed in studies on both DT and sarcoma populations [13,15,18]. These findings underline that HRQoL in DT is not only determined by tumor biology but also by the functional and psychosocial context of the tumor’s anatomical location, suggesting that rehabilitation and psychosocial support should be tailored accordingly.

Overall, HRQoL in DT is influenced by multiple interacting factors rather than by disease activity alone. Female gender and unemployment consistently predicted lower HRQoL, reflecting psychosocial vulnerability and the stabilizing role of work participation. Intensive or multimodal treatment was linked to persistent physical and symptomatic burden, while time since diagnosis or treatment showed little improvement over time, pointing to the chronic nature of the disease. Tumor site, particularly lesions of the lower extremities, added further heterogeneity through mobility restrictions and body image concerns. Together, these findings emphasize that HRQoL in DT patients is shaped by both medical and psychosocial dimensions, requiring integrated long-term care that supports physical recovery and emotional adaptation alike.

### 4.6. Clinical and Psychosocial Implications

The results underline that desmoid-type fibromatosis requires a chronic-care perspective rather than short-term oncological follow-up. Persistent pain, fatigue, and emotional distress call for structured long-term programs that integrate symptom control, psychological support, and physical rehabilitation. Given the strong influence of gender and employment status, interventions should include gender-sensitive counseling and vocational reintegration support. Routine assessment of HRQoL with instruments such as the DTF-QoL or EORTC QLQ-C30 can help identify patients at risk for long-term distress and guide individualized supportive care. Overall, multidisciplinary coordination between oncology, psycho-oncology, and rehabilitation services is essential to sustain quality of life beyond tumor management.

### 4.7. Strengths and Limitations

Major strengths of this study include the comparatively large DT cohort, the use of both generic and disease-specific HRQoL instruments, and the integration of reference data from sarcoma and international DT populations. These features provide a broad contextual understanding of quality of life in this rare disease. However, the cross-sectional design limits causal interpretation. Recruitment through patient organizations could have led to selection bias toward more engaged or long-term survivors. Likewise, most hospital-based participants were recruited from a single specialized center, which may limit representativeness. A further potential source of bias concerns educational level, as patients with higher education are typically overrepresented in observational studies of this kind. Moreover, when comparing our DTF cohort with external datasets, standardization for sex and gender was not possible, which may have influenced between-cohort comparisons. Finally, because the majority of participants were long-term patients, recent therapeutic developments—such as the growing use of active surveillance strategies—are not fully reflected in this dataset. In addition, linear regression models assume approximately normally distributed residuals. Although HRQoL scales may deviate from normality, linear regression is widely used in analyses of EORTC QLQ-C30 outcomes and provides interpretable estimates of mean differences between patient groups.

## 5. Conclusions

Desmoid-type fibromatosis causes a sustained reduction in health-related quality of life, in some aspects comparable in magnitude to that of malignant sarcomas. HRQoL is determined not by disease activity alone, but by psychosocial and treatment-related factors—particularly gender, employment status, treatment intensity, and tumor site. As improvements over time remain limited, DT should be regarded as a chronic condition requiring long-term, multidisciplinary care. Integrating routine HRQoL assessment and individualized support into clinical follow-up can help preserve functioning and well-being in this rare tumor population.

## Figures and Tables

**Table 1:** Description study population. Comparison of patients from the PROSa and the PROSa-DES studies.

Variable	Complete Dataset		PROSa-Desmoid		PROSa	
Value	N	%	N	%	N	%
All	155		109	70.3	46	29.7
Age Groups						
18–35	45	29.6	29	26.9	16	36.4
36–55	74	48.7	57	52.8	17	38.6
>55	33	21.7	22	20.4	11	25
Age (mean. SD)	45.0	14.3	45.7	14.1	43.4	14.8
Gender						
female	108	69.7	76	69.7	32	69.6
male	45	29	31	28.4	14	30.4
diverse	2	1.3	2	1.8	0	0
Time since diagnosis						
0–<5 year	64	41.6	29	26.6	35	77.8
5–<10 year	34	22.1	31	28.4	3	6.7
>10 years	56	36.4	49	45	7	15.6
Time since diagnosis (mean. SD)	7.9	6.4	9.5	6.1	4.0	5.4
Time since treatment						
in treatment			17	17.9		
0–<5 years			28	25.7		
5–<10 years			30	31.6		
more than 10 years			20	21.1		
Time since treatment (mean. SD)			5.8	4.7		
Medical lines						
0 to 1			83	53.5		
2 or more			23	14.8		
unknown			3	1.9		
Recurrence after Surgery						
no	52	33.5	33	30.3	19	41.3
yes	60	38.7	43	39.4	17	37
unknown or no OP	43	27.7	33	30.3	10	21.7
Tumor Location						
Lower extremity (incl hip/pelvis)	48	31	32	29.4	16	34.8
Shoulder and upper extremity	12	7.7	8	7.3	4	8.7
Trunk (thoracic wall and back)	27	17.4	20	18.3	7	15.2
Abdominal wall	17	11	17	15.6	0	0
Intra-abdominal	36	23.2	24	22	12	26.1
Head and neck	8	5.2	7	6.4	1	2.2
not defined/multifocal	7	4.5	1	0.9	6	13
Received treatments						
surgery only			30	27.5		
ST only			14	12.8		
ST + surgery			19	17.4		
ST + RT + surgery			21	19.3		
none (W&W)			7	6.4		
all other			18	16.5		
Education						
basic/medium	58	37.7	36	33	22	48.9
high	96	62.3	73	67	23	51.1
Status Employment						
employed	114	73.5	78	71.6	36	78.3
unemployed. disability pension	17	11	14	12.8	3	6.5
retirement pension	15	9.7	12	11	3	6.5
other	9	5.8	5	4.6	4	8.7

**Table 2:** Factors associated with the DTF QoL Scales. Multivariable Regression. B = unstandardized regression coefficient. *p* = *p*-value. ST = systemic therapy. RT = radiotherapy. * Others not shown. Significant differences bold.

Desmoid-Type Fibromatosis Quality of Life Questionnaire (DTF QoL)														
	Symptom Scales B (p)			Impact Scales B (p)										
	W1 Emotional Consequences	W2 Physical Consequences	W3 Pain and Discomfort	1 Concerns Condition	2 Job and Education	3 Doctor–Patient Relationship	4 Effect of DTF on Relationships	5 Physical Limitations and Consequences	6 Diagnostic and Treatment Trajectory	7 Parenting and Fertility	8 Body Image and Sensation	9 Supportive Care	10 Concerns Around Treatment and Its Consequences	11 Unpredictable Course and Nature of DTF
Age Groups														
18–35 years (ref)														
36–55 years	/	/	/	−1.2 (0.81)	/	/	/	/	/	**−43.2** **(0.00)**	2.5 (0.65)	/	/	/
>55 years	/	/	/	−11.3 (0.07)	/	/	/	/	/	−6.5 (0.40)	−8.5 (0.22)	/	/	/
Gender *														
woman (ref)														
men	**−11.9** **(<0.01)**	**−12.2** **(<0.01)**	**−15.7** **(<0.01)**	**−10.4** **(0.03)**	/	**−17.6** **(<0.001)**	**−19.2** **(<0.001)**	**−13.5** **(<0.01)**	**−13.0** **(<0.001)**	**−26.6** **(<0.001)**	**−14.7** **(<0.01)**	**−16.3** **(<0.001)**	**−15.4** **(<0.01)**	**−14.9** **(<0.001)**
Time since treatment														
In treatment (ref)														
0–<5 years	/	/	/	/	/	/	/	/	/	/	/	/	/	/
5–<10 years	/	/	/	/	/	/	/	/	/	/	/	/	/	/
>10 years	/	/	/	/	/	/	/	/	/	/	/	/	/	/
Time since diagnosis														
0–<5 years (ref.)														
5–<10 years	/	**11.5** **(0.03)**	/	/	/	/	/	/	/	/	/	−6.1 (0.31)	/	/
>10 years	/	1.7 (0.73)	/	/	/	/	/	/	/	/	/	−8.9 (0.12)	/	/
No of ST lines														
0 or 1 line (ref.)														
≥2 lines	7.3 (0.11)	/	**19.0** **(<0.01)**	7.7 (0.14)	12.3 (0.09)	/	5.5 (0.24)	8.4 (0.09)	/	/	**15.3** **(<0.01)**	/	9.7 (0.07)	**13.9** **(<0.01)**
Recurrence *														
no (ref.)														
yes	/	/	/	/	/	/	/	/	/	−13.3 (0.10)	/	**−11.4** **(0.03)**	/	/
Tumor location *														
Lower extremity (incl hip/pelvis) (ref.)														
Upper extremity	/	**−17.2** **(0.02)**	6.3 (0.46)	/	/	/	/	/	/	**−46.5** **(<0.01)**	/	/	**−24.3** **(<0.01)**	/
Trunk (thoracic wall and back)	/	**−13.0** **(0.02)**	−10.1 (0.12)	/	/	/	/	/	/	−17.2 (0.09)	/	/	−7.0 (0.23)	/
Abdominal wall	/	**−16.0** **(<0.01)**	−12.4 (0.07)	/	/	/	/	/	/	−10.2 (0.29)	/	/	**−14.1** **(0.02)**	/
Intra-abdominal	/	−10.6 (0.06)	−8.2 (0.20)	/	/	/	/	/	/	1.2 (0.90)	/	/	−7.7 (0.19)	/
Head and Neck	/	**−18.2** **(0.02)**	−4.3 (0.64)	/	/	/	/	/	/	−30.5 (0.06)	/	/	**−26.6** **(<0.01)**	/
Received treatments *														
Surgery and/or ST (ref.)														
ST + RT + surgery	/	**11.9** **(0.02)**	9.9 (0.09)	7.3 (0.18)	/	/	/	**11.8** **(0.02)**	7.4 (0.10)	/	9.1 (0.13)	/	**11.5** **(0.03)**	6.8 (0.16)
Education														
basic/medium (ref.)														
high	/	/	/	/	/	/	/	/	/	/	/	/	/	/
Status Employment *														
employed (ref.)														
unemployed, disability pension	**26.0** **(<0.001)**	**26.7** **(<0.001)**	**16.9** **(0.01)**	/	**44.5** **(<0.001)**	/	**25.4** **(<0.001)**	**20.9** **(<0.001)**	/	/	/	/	**18.8** **(<0.01)**	/
retirement pension	5.8 (0.33)	11.3 (0.05)	−1.1 (0.87)	/	16.8 (0.20)	/	5.0 (0.40)	9.4 (0.13)	/	/	/	/	−1.5 (0.83)	/

**Table 3:** Factors associated with Scales of the EORTC QLQ-C30. B = unstandardized regression coefficient. *p* = *p*-value. ST = systemic therapy. RT = radio therapy. * Others not shown. Significant differences bold.

European Organisation for Research and Treatment of Cancer Quality of Life Core Questionnaire (EORTC QLQ-C30)															
	Functional Scales B (p)						Symptom Scales B (p)								
	PF (N = 109)	RF (N = 109)	EF (N = 154)	CF (N = 109)	SF (N = 154)	Global QoL (N = 107)	FA (N = 109)	NV (N = 151)	Pain (N = 109)	Dyspnea (N = 153)	Insomnia (N = 154)	Appetite Loss (N = 155)	Constipation (N = 154)	Diarrhea (N = 109)	Financial Diff (N = 152)
Age Groups															
18–35 years (ref)															
36–55 years	/	/	/	/	/	/	/	**−6.1 (0.01)**	/	−7.6 (0.11)	/	/	/	/	/
>55 years	/	/	/	/	/	/	/	**−12.6 (<0.001)**	/	−0.5 (0.94)	/	/	/	/	/
Gender *															
woman (ref)															
men	**13.1 (<0.001)**	/	**16.2 (<0.001)**	/	/	**12.4 (<0.01)**	/	/	**−23.2 (<0.001)**	/	/	/	/	/	/
Time since treatment															
In treatment (ref)															
0–<5 years	/	/	/	2.3 (0.77)	/	−3.9 (0.46)	−4.3 (0.60)	/	/	/	/	/	/	/	/
5–<10 years	/	/	/	6.2 (0.42)	/	−0.9 (0.87)	−6.2 (0.45)	/	/	/	/	/	/	/	/
>10 years	/	/	/	**20.0 (0.02)**	/	7.7 (0.19)	**−17.9 (0.04)**	/	/	/	/	/	/	/	/
Time since diagnosis															
0–<5 years (ref.)															
5–<10 years	/	/	/	/	/	/	/	2.2 (0.41)	/	/	−3.5 (0.60)	/	/	3.5 (0.62)	/
>10 years	/	/	/	/	/	/	/	−3.1 (0.19)	/	/	**−12.2 (0.03)**	/	/	−10.7 (0.09)	/
No. of ST lines															
0 or 1 line (ref.)															
≥2 lines	/	/	/	/	/	/	/	/	12.0 (0.07)	/	/	/	/	**13.4 (0.04)**	/
Recurrence *															
no (ref.)															
yes	/	/	/	/	/	/	/	/	/	/	/	/	/	/	/
Tumor location *															
Lower extremity (incl hip/pelvis) (ref.)															
Upper extremity	9.9 (0.13)	/	/	/	/	/	/	/	/	/	/	/	/	/	/
Trunk (thoracic wall and back)	**12.8 (0.01)**	/	/	/	/	/	/	/	/	/	/	/	/	/	/
Abdominal wall	**11.4 (0.03)**	/	/	/	/	/	/	/	/	/	/	/	/	/	/
Intra-abdominal	1.8 (0.71)	/	/	/	/	/	/	/	/	/	/	/	/	/	/
Head and Neck	5.5 (0.42)	/	/	/	/	/	/	/	/	/	/	/	/	/	/
Received treatments *															
surgery and/or ST (ref.)															
ST + RT + surgery	**−15.3 (<0.001)**	−12.9 (0.07)	/	/	/	**−9.6 (0.02)**	12.1 (0.07)	/	**13.9 (0.04)**	/	/	/	/	/	/
Education															
basic/medium (ref.)															
high	/	/	/	/	/	/	/	/	/	/	/	/	8.4 (0.05)	−8.8 (0.15)	/
Status Employment *															
employed (ref.)															
unemployed, disability pension	**−27.4 (<0.001)**	**−33.0 (<0.001)**	**−23.8 (<0.001)**	**−34.9 (<0.001)**	**−40.3 (<0.001)**	**−25.3 (<0.001)**	**31.8 (<0.001)**	**15.3 (<0.001)**	**33.5 (<0.001)**	**12.7 (0.07)**	**39.6 (<0.001)**	**14.3 (<0.01)**	/	/	**48.5 (<0.001)**
retirement pension	−4.4 (0.41)	−10.0 (0.25)	2.9 (0.68)	−5.2 (0.51)	−6.9 (0.38)	−3.5 (0.52)	1.7 (0.83)	6.8 (0.12)	−0.6 (0.94)	9.7 (0.26)	8.7 (0.31)	3.8 (0.46)	/	/	10.5 (0.18)

## Data Availability

The data presented in this study are not publicly available due to privacy and ethical restrictions, but are available from the corresponding author upon reasonable request.

## Supplementary Materials

The following supporting information can be downloaded at: https://www.mdpi.com/article/10.3390/cancers18061046/s1, Table S1: Factors associated with the DTF QoL Scales. Table S2: Factors associated with Scales of the EORTC QLQ-C30. Table S3: Distribution of EORTC QLQ-C30 scores in the study population (N = 155) and results of Shapiro–Wilk normality testing. Table S4: Distribution of DTF-QoL scores in the PROSa-DES study population (N = 109) and results of Shapiro–Wilk normality testing. Table S5: Model fit statistics of the generalized linear models for DTF-QoL and EORTC QLQ-C30 outcomes.
