# Supplementary material for: The Health-Related Quality of Life of German Desmoid Patients: Results from the PROSa-DES and PROSa Study
Source: Cancers (Basel). 2026 Mar 23;18(6):1046. doi: 10.3390/cancers18061046 (PMC13025788; doi:10.3390/cancers18061046)
Supplement: Supplementary file 1 [file cancers-18-01046-s001.zip › cancers-4193776-supplementary.pdf]

Supplementary

Supplementary Table S1: Distribution of EORTC QLQ-C30 scores in the study population (N = 155) and results of Shapiro–Wilk normality testing. SD= standard deviation.

Scale	N	Mean	SD	Shapiro–Wilk W	p-value
Physical Functioning	155	80.4	21.3	0.84	<.001
Role Functioning	155	65.8	30.8	0.89	<.001
Emotional Functioning	155	56.7	27.9	0.96	<.001
Cognitive Functioning	155	76.4	27.6	0.81	<.001
Social Functioning	155	68.8	31.4	0.86	<.001
Global Health Status / QoL	155	61.8	20.9	0.96	<.001
Fatigue	155	40.1	29.5	0.94	<.001
Nausea / Vomiting	155	5.8	13.6	0.50	<.001
Pain	155	38.0	32.2	0.90	<.001
Dyspnea	155	16.8	26.1	0.67	<.001
Insomnia	155	38.5	34.4	0.85	<.001
Appetite Loss	155	9.2	19.2	0.53	<.001
Constipation	155	12.3	26.3	0.51	<.001
Diarrhea	155	16.3	25.9	0.66	<.001
Financial Difficulties	155	20.4	32.3	0.66	<.001

Supplementary Table S2: Distribution of DTF-QoL scores in the PROSa-DES study population (N = 109) and results of Shapiro–Wilk normality testing. SD= standard deviation.

Scale	N	Mean	SD	Shapiro–Wilk W	p-value
Emotional consequences	109	27.0	21.3	0.92	<.01
Physical consequences	109	19.1	23.6	0.76	<.001
Pain and discomfort	109	27.0	27.2	0.85	<.001
Concerns about condition	109	64.4	22.4	0.95	.02
Job and education	109	47.0	33.2	0.93	<.01
Doctor–patient relationship	109	39.1	24.4	0.94	.02
Effect on relationships	109	39.3	22.3	0.97	.33
Physical limitations	109	36.0	22.6	0.96	.01
Diagnostic and treatment trajectory	109	51.0	18.9	0.97	.21
Parenting and fertility	109	52.0	30.1	0.95	.03
Body image and sensation	109	43.0	25.5	0.97	.21
Supportive care	109	38.1	25.1	0.97	.28

Scale	N	Mean	SD	Shapiro–Wilk W	p-value
Treatment-related concerns	109	44.3	24.8	0.96	.054
Unpredictable course of disease	109	56.0	20.8	0.99	.85

Supplementary Table S3: Model fit statistics of the generalized linear models for DTF-QoL and EORTC QLQ-C30 outcomes.

Outcome	Likelihood Ratio χ^2	df	p-value
**DTF-QoL**			
Emotional consequences	29.9	7	<.001
Physical consequences	54.8	15	<.001
Pain and discomfort	54.0	15	<.001
Concerns about condition	19.9	8	.01
Job and education	31.4	7	<.001
Doctor–patient relationship	12.2	2	<.01
Effect on relationships	37.9	7	<.001
Physical limitations and consequences	34.2	9	<.001
Diagnostic and treatment trajectory	14.5	4	<.01
Parenting and fertility	40.0	10	<.001
Body image and sensation	27.2	8	<.001
Supportive care	26.1	6	<.001
Concerns around treatment and its consequences	52.0	15	<.001
Unpredictable course and nature of DTF	54.0	15	<.001
**EORTC QLQ-C30**			
Physical Functioning	59.8	13	<.001
Role Functioning	23.7	5	<.001
Emotional Functioning	22.1	5	<.001
Cognitive Functioning	38.4	7	<.001
Social Functioning	28.1	3	<.001
Global QoL	41.5	11	<.001
Fatigue	33.2	9	<.001
Nausea/Vomiting	30.6	7	<.001
Pain	44.7	9	<.001
Dyspnea	9.5	5	.01
Insomnia	26.7	5	<.001

Outcome	Likelihood Ratio χ^2	df	p-value
Appetite Loss	8.7	3	.03
Constipation	3.7	1	.054
Diarrhea	12.7	5	.03
Financial Difficulties	35.9	3	<.001

*Supplementary Table S4: Factors associated with the DTF QoL Scales. Univariate analyses. p= p-value. SD= standard deviation. ST= systemic therapy. RT= radio therapy. *Included in multivariable model. **T-test. ***Anova. Significant differences bold.*

	**Desmoid-Type Fibromatosis Quality of Life Questionnaire (DTF QoL)**													
	**Symptom Scales**			**Impact Scales**										
	**W1 Emotional consequences**	**W2 Physical consequences**	**W3 Pain and discomfort**	**1 Concerns about condition**	**2 Job and education**	**3 Doctor–patient relationship**	**4 Effect of DTF on relationships**	**5 Physical limitations and consequences**	**6 Diagnostic and treatment trajectory**	**7 Parenting and fertility**	**8 Body image and sensation**	**9 Supportive care**	**10 Concerns around treatment**	**11 Unpredictable course and nature of DTF**
**Study Population (N=109)**														
Mean	27,0	19,1	27,0	64,4	47,0	39.1	39,3	36,0	51,0	52,0	43,0	38,1	44,3	56,0
SD	21,3	23,6	27,2	22,4	33,2	24,4	22,3	22,6	18,9	30,1	25,5	25,1	24,8	20,8
**Comparison**														
Shut 2022 (N=235)	15,3	11,6	19,6	41,3	29,2	26,7	24,8	18,7	28,1	21,1	29,0	36,0	22,1	38,8
SD	18,7	16,5	25,6	24,1	31,6	19,7	21,8	18,9	19,1	23,1	25,9	14,1	21,4	22,4
**Age Groups**														
18-35 years	27,7	22,1	27,2	68,7	45,3	37,9	41,1	37,7	51,6	59,8	46,0	32,0	48,8	58,1
SD	23,4	27,2	29,9	19,2	32,0	24,2	22,7	24,8	19,4	32,3	26,5	27,0	27,9	19,9
36-55 years	27,4	16,4	28,7	66,7	47,1	41,5	41,3	34,6	53,0	54,0	46,1	41,5	43,8	57,2
SD	21,0	21,9	26,6	22,4	33,3	25,0	22,1	20,3	18,1	27,3	24,7	25,8	21,0	20,1
> 55 years	24,8	21,8	22,2	53,2	46,5	33,5	31,7	36,2	44,3	10,0	31,2	36,1	36,9	48,8
SD	20,4	23,6	26,3	23,9	36,1	23,4	22,2	26,0	19,7	15,9	24,3	18,6	27,4	22,7
p***	0,87	0,48	0,65	**0,03***	0,97	0,41	0,20	0,83	0,18	**0,01***	*0,05**	0,23	0,24	0,21

**Gender**														
woman	30,0	22,3	32,0	68,3	47,9	44,3	44,4	38,6	54,9	59,8	48,4	43,9	47,8	60,5
SD	20,4	24,7	27,1	21,4	34,2	25,4	22,0	23,2	18,8	27,6	25,2	24,6	24,4	20,6
men	20,0	12,5	15,8	56,2	43,8	26,7	27,2	28,2	41,8	28,6	30,8	24,6	35,6	45,7
SD	22,7	19,7	24,8	23,0	32,1	17,6	18,8	19,5	16,7	25,6	22,4	20,3	24,9	17,8
p**	0,03*	0,05*	<,01*	0,01*	0,60	<,001*	<,001*	0,02*	<,001*	<,001*	<,01*	<,001*	0,02*	<,01*
**Time since treatment**														
in treatment	26,0	22,4	37,3	59,6	54,6	35,1	34,8	35,5	49,4	34,4	40,5	36,3	45,5	57,5
SD	19,6	24,8	30,2	20,8	37,6	25,3	21,8	22,3	15,7	44,5	30,1	31,8	26,0	18,9
0–<5 years	29,9	18,5	25,4	64,1	46,5	41,1	39,5	35,8	47,8	47,1	45,1	35,7	40,3	56,5
SD	20,8	25,3	26,9	21,3	32,0	25,7	22,6	23,0	16,7	33,0	23,3	23,5	20,5	21,3
5–<10 years	26,2	22,2	28,1	66,7	47,5	35,6	43,3	36,8	50,8	54,3	43,6	43,3	45,1	56,6
SD	21,9	28,4	27,9	22,3	36,5	24,4	23,2	22,9	21,6	24,7	28,8	23,0	28,6	21,2
>10 years	17,9	11,7	15,6	59,0	34,1	41,6	32,1	33,7	48,5	42,8	42,8	28,6	38,2	47,4
SD	14,8	12,4	20,5	24,6	24,6	22,8	21,9	22,6	19,7	34,0	24,2	22,0	18,0	21,3
p***	0,23	0,45	0,11	0,58	0,38	0,72	0,30	0,97	0,94	0,63	0,95	0,23	0,69	0,22
**Time since diagnosis**														
0–<5 years	28,0	12,2	19,9	62,5	50,4	48,6	42,0	31,1	53,0	56,5	35,2	47,1	41,7	55,3
SD	21,6	13,5	22,3	22,8	31,3	23,4	24,0	22,1	19,8	33,4	21,6	26,7	25,3	20,3
5–<10 years	32,7	26,7	33,0	65,2	53,4	38,5	40,0	38,5	52,6	44,5	46,6	37,5	44,9	57,6
SD	25,8	29,1	31,6	24,4	38,7	29,0	23,5	25,3	20,2	30,5	29,8	25,6	27,6	24,4
>10 years	22,9	18,5	27,4	65,1	40,9	33,9	37,2	37,2	48,8	52,5	45,4	33,2	45,4	55,4
SD	17,2	23,5	26,5	21,2	29,8	20,4	20,7	21,2	17,7	28,0	24,2	22,7	22,9	18,9
p***	0,13	0,06*	0,18	0,86	0,25	0,04*	0,64	0,40	0,55	0,58	0,16	0,06*	0,81	0,89
**No. of systemic treatments (N=95)**														
0 or 1 line	24,4	15,5	20,9	62,5	42,4	38,7	37,3	32,6	50,9	52,8	39,4	37,7	39,9	52,6
SD	20,1	22,1	24,6	22,1	31,9	24,9	22,5	21,1	19,6	30,3	25,0	24,9	22,5	20,4

≥2 lines	36,1	30,8	47,3	73,0	61,0	41,8	47,6	47,5	52,0	53,1	56,8	37,0	59,3	68,0
SD	23,9	24,9	26,2	21,4	33,4	23,4	20,5	23,9	16,6	28,9	24,3	26,5	24,9	17,3
p**	**0,02***	**<,01***	**<,001***	**0,04***	**0,02***	0,59	**0,04***	**<,01***	0,80	0,98	**<,01***	0,90	**<,001***	**<,01***
**Recurrence (surgery only N =76)**														
no	28,5	18,3	23,2	64,9	45,4	43,9	40,4	34,9	52,4	59,2	45,7	43,6	43,0	54,9
SD	22,9	21,7	26,6	21,3	32,3	25,8	22,5	21,0	16,5	28,9	23,9	24,6	19,0	20,0
yes	26,6	21,0	29,5	62,9	46,1	32,4	36,2	35,9	48,2	42,7	44,7	28,3	47,7	54,1
SD	22,4	26,2	29,6	21,7	34,3	23,4	20,5	23,8	17,9	27,7	27,8	23,5	27,5	21,5
p**	0,71	0,64	0,35	0,69	0,93	**0,04***	0,40	0,85	0,30	_0,08_*	0,87	**<,01***	0,39	0,86
**Tumor location**														
Lower extremity (incl Hip/ pelvis)	31,8	31,7	38,5	68,5	45,3	36,5	42,0	38,4	50,6	58,2	45,9	41,3	56,5	59,4
SD	24,2	31,7	32,9	20,6	35,6	25,0	20,5	24,1	20,9	29,9	27,5	27,1	26,5	21,8
Upper Extremity (incl shoulder)	22,4	18,8	41,7	57,3	45,6	30,6	34,5	36,1	52,2	13,3	41,3	34,0	33,0	54,7
SD	18,4	22,1	28,3	18,7	40,1	20,4	19,1	25,0	17,9	23,1	28,3	21,1	22,0	21,5
Trunk (thoracic wall & back)	21,3	14,7	22,8	53,8	39,6	35,3	32,6	30,8	51,5	33,7	43,5	30,3	43,5	52,1
SD	16,5	18,4	20,9	22,5	28,7	22,4	17,3	23,5	18,9	22,0	22,1	19,5	22,2	20,1
Abdominal wall	23,8	11,0	17,0	68,6	38,2	44,3	35,1	28,0	55,0	57,2	47,2	33,3	38,9	54,5
SD	21,1	15,3	20,8	21,2	29,7	28,2	24,2	18,6	12,2	23,5	22,2	25,2	15,9	18,7
Intra-abdominal	29,2	11,9	16,7	69,8	58,0	46,1	45,4	40,9	47,7	60,1	40,2	45,4	38,1	56,7
SD	22,3	14,3	22,7	22,9	30,7	22,3	28,1	22,5	21,2	32,9	29,1	23,8	25,4	19,8
Head & Neck	26,8	18,1	28,6	56,5	56,7	32,4	41,0	38,8	49,2	43,1	30,2	33,3	30,2	50,3
SD	22,2	21,0	23,0	28,9	38,8	28,9	20,8	19,5	19,5	49,1	21,2	34,5	20,0	28,0
p***	0,67	**0,02***	**0,02***	0,16	0,30	0,44	0,54	0,47	0,85	_0,08_*	0,82	0,34	**<,01***	0,72
**Received treatments N=84**														
Surgery and/ or ST	25,1	15,1	20,6	62,4	44,7	39,8	37,9	32,4	48,2	51,0	39,4	38,7	40,2	53,3
SD	20,4	18,7	23,2	22,9	31,4	25,3	22,6	20,1	19,5	29,8	24,4	24,7	25,3	20,5
Surgery + ST + RT	30,4	31,9	41,3	69,6	51,1	33,1	43,1	47,1	54,8	46,7	50,1	32,5	53,5	62,9

SD	22,9	28,2	32,2	15,0	38,5	23,7	20,8	25,3	15,1	26,2	21,0	27,8	20,2	16,8
p**	0,33	**0,02***	**0,01***	_0,10_	0,48	0,29	0,35	**<,01***	0,16	0,76	_0,08_*	0,34	**0,04***	_0,06_*
**Education**														
basic/ medium	30,9	20,8	32,4	63,9	55,3	35,3	42,9	39,5	49,9	45,6	42,6	41,0	45,6	56,5
SD	24,1	23,6	26,7	24,8	35,9	22,5	24,6	24,2	16,5	31,8	26,2	25,9	26,7	19,0
high	25,1	18,3	24,4	64,7	42,9	41,0	37,5	34,2	51,6	55,1	43,2	36,7	43,6	55,7
SD	19,7	23,7	27,3	21,3	31,3	25,3	21,0	21,7	20,1	29,3	25,3	24,7	24,1	21,7
p**	0,18	0,60	0,15	0,86	_0,09*_	0,26	0,23	0,25	0,67	0,29	0,90	0,40	0,71	0,85
**Status Employment**														
employed	23,4	14,3	24,4	63,6	41,5	37,3	35,9	32,8	50,4	50,6	42,0	36,4	42,7	54,3
SD	19,1	21,1	25,8	20,9	29,5	23,8	20,3	20,9	18,7	28,8	24,9	26,0	22,5	19,3
unemployed, disability pension	48,2	43,3	49,2	74,1	85,1	41,3	57,1	53,8	54,9	50,2	51,8	47,6	64,7	66,4
SD	23,4	19,2	27,8	26,8	26,0	25,4	22,0	19,0	14,6	36,5	24,7	23,6	25,7	18,8
retirement pension	26,0	24,4	20,4	59,7	58,7	45,2	36,7	39,3	51,2	na	40,5	38,4	36,6	55,4
SD	21,0	29,2	25,0	25,3	40,9	25,0	24,1	28,7	22,8	na	29,6	16,3	25,0	27,3
other	26,7	14,7	22,2	62,5	25,3	47,8	48,2	28,1	49,5	75,0	40,0	37,8	32,2	55,2
SD	21,0	16,6	31,4	24,5	33,5	33,5	29,7	22,5	27,5	36,3	30,3	32,0	34,8	29,0
p***	**<,001***	**<,001***	**0,01***	0,35	**<,001***	0,59	**<,01***	**<,01***	0,88	0,40	0,59	0,50	**<,01***	0,25

*Supplementary Table S5: Factors associated with Scales of the EORTC QLQ-C30. Univariate analyses. p= p-value. SD= standard deviation. ST= systemic therapy. RT= radio therapy. PF= physical functioning. RF= role functioning. EF= emotional functioning. CF= cognitive functioning. SF= social functioning. FD= financial difficulties. *Included in multivariable model. **T-test. ***Anova. Significant differences bold.*

	**European Organisation for Research and Treatment of Cancer Quality of Life Core Questionnaire (EORTC QLQ-C30)**														
	**Functional Scales**						**Symptom Scales**								
	**PF**	**RF**	**EF**	**CF**	**SF**	**Global QoL**	**Fatigue**	**Nausea**	**Pain**	**Dyspnea**	**Insomnia**	**Appetite Loss**	**Constipation**	**Diarrhea**	**FD**
**Study Population**															
Study Population N=155 mean	80.4	65.8	56.7	76.4	68.8	61.8	40.1	5.8	38.0	16.8	38.5	9.2	12.3	16.3	20.4
SD	21.3	30.8	27.9	27.6	31.4	20.9	29.5	13.6	32.2	26.1	34.4	19.2	26.3	25.9	32.3
PROSa - Desmoid population N=109 mean	80.0	65.6	56.9	74.2	70.2	61.6	41.6	6.9	37.2	18.0	38.5	10.4	12.5	17.7	20.4
SD	21.6	30.7	28.9	29.0	31.2	20.4	29.6	14.9	31.2	27.4	34.0	21.1	27.5	27.8	33.1
PROSa Desmoid Patients N=46 mean	81.3	66.3	56.2	81.9	65.6	62.3	36.5	3.3	39.9	13.8	38.4	6.5	11.6	13.0	20.5
SD	20.8	31.3	25.6	23.5	31.7	22.4	29.1	9.7	34.5	22.8	35.8	13.4	23.5	20.5	30.7
p**	0.72	0.90	0.88	0.11	0.40	0.85	0.33	0.13	0.63	0.35	0.98	0.25	0.84	0.30	0.99
**Comparisons**															
German Population Nolte 2019	82.8	80.8	73.9	83.9	84.8	67.0	31.5	6.0	27.6	18.7	27.6	10.1	9.6	10.4	11.3
SD	21.2	27.2	24.7	22.7	25.5	21.8	27.2	17.2	30.9	27.3	33.1	23.3	22.3	22.7	25.0
Sarcoma Patients Eichler 2020 N=1096	72.2	54.1	60.0	77.3	57.8	59.4	43.2	9.0	34.0	25.5	38.5	18.1	16.5	13.3	25.8
SD	24.4	33.6	26.5	25.3	33.1	22.7	28.5	18.4	31.6	30.1	34.1	28.4	28.6	25.3	33.2
Shut 2022 N= 235	86.1	82.1	79.3	84.8	83.8	76.3	23.0	3.2	22.5	8.8	25.4	6.0	10.9	8.9	10.5
SD	18.6	27.0	21.3	21.2	26.0	19.6	24.9	8.9	26.5	19.2	31.6	16.4	21.3	21.1	25.3
Garg 2022 N=105	76.2	72.9	67.6	87.1	79.1	65.6	31.2	5.7	34.6	6.5	21.9	12.7	9.5	3.9	29.1
SD	19.4	27.3	24.0	20.2	26.9	22.6	24.1	12.2	28.3	16.3	28.3	22.5	17.1	14.2	35.0
**Age Groups**															
18-35 years	81.1	65.6	53.1	78.5	71.1	63	42.2	10.4	43.3	18.5	34.8	11.8	14.1	17.8	21.5
SD	23.0	34.2	28.8	27.9	31.9	23.3	32.2	18.9	34.0	29.8	35.5	20.3	27.1	28.1	33.5
36-55 years	82.9	70.7	56.5	78.4	71.2	62.6	37.5	4.5	34.7	11.7	37.8	8.1	12.2	17.6	18.5
SD	18.2	27.0	28.2	26.4	28.8	18.0	27.6	11.5	28.8	21.7	33.7	19.7	26.2	26.6	32.1
> 55 years	75.0	58.6	63.6	72.2	60.6	59.6	39.4	3.0	35.4	24.2	44.4	8.1	10.1	11.1	22.9
SD	25.0	31.8	25.6	26.9	36.5	24.0	29.4	7.7	36.0	28.0	34.0	16.7	27.0	21.5	32.2
p***	0.20	0.16	0.26	0.51	0.24	0.76	0.70	**0.03***	0.33	*0.06**	0.47	0.55	0.81	0.45	0.79

**Gender**															
woman	78.3	63.7	52.4	74.5	67.0	59.4	42.6	6.6	43.8	17.0	41.4	9.6	14.2	16.0	21.6
SD	20.8	31.6	27.3	29.0	30.7	20.6	29.9	15.3	31.7	26.4	35.0	19.9	28.2	25.2	32.3
men	85.2	70.7	66.2	80.7	73.1	67.7	34.1	4.1	25.2	16.3	32.6	8.1	8.1	17.0	18.3
SD	22.4	29.1	27.4	24.6	33.4	21.3	28.5	8.8	29.7	26.2	32.9	17.6	21.5	28.1	33.1
p**	*0.07*	0.20	**0.01***	0.21	0.28	**0.03***	0.11	0.20	**<.001***	0.88	0.15	0.68	0.15	0.83	0.57
**Time since treatment N=95**															
in treatment	80.7	67.6	60.1	**67.6**	73.5	61.3	50.3	8.8	45.1	11.8	39.2	9.8	19.6	21.6	13.7
SD	20.6	33.1	28.1	30.9	28.9	21.8	30.5	20.5	32.1	28.7	37.7	15.7	35.5	31.0	29.0
0–<5 years	75.2	64.3	54.8	69.0	66.7	57.1	44.4	10.1	38.1	20.2	40.5	7.1	10.7	17.9	17.9
SD	25.5	31.0	28.2	28.2	30.4	18.7	26.7	18.3	29.0	24.6	33.2	13.9	15.9	24.8	29.4
5–<10 years	79.6	66.7	54.4	75.6	73.3	60.8	39.3	6.1	37.8	16.7	37.8	11.1	14.4	20.0	28.9
SD	19.2	29.4	27.4	28.3	29.2	23.6	30.0	12.0	34.7	27.3	32.4	23.7	32.4	29.8	37.9
>10 years	88.3	68.3	68.9	92.5	76.3	74.1	26.1	0.0	21.7	20.0	21.7	6.7	5.0	10.0	8.8
SD	14.0	28.0	25.7	11.4	33.5	12.1	20.5	0.0	21.0	27.4	22.4	17.4	22.4	19.0	21.8
p***	0.20	0.97	0.27	**0.01***	0.72	**0.05***	**0.05***	0.12	0.11	0.74	0.19	0.80	0.40	0.53	0.14
**Time since diagnosis**															
0–<5 years – MW SD	82.5	66.7	55.7	74.7	70.7	60.9	39.8	9.8	32.8	13.8	48.3	13.8	10.3	19.5	18.4
SD	17.0	29.2	31.5	29.4	27.0	15.9	30.9	16.4	27.6	20.9	35.2	22.7	23.7	32.8	29.0
5–<10 years – MW SD	75.9	58.6	52.3	61.8	65.6	57.3	50.5	10.8	46.2	25.8	44.1	11.8	15.1	25.8	30.1
SD	26.0	34.4	32.6	34.7	35.5	26.2	34.0	20.4	36.2	35.2	38.9	26.6	28.3	31.9	43.3
>10 years – MW SD	81.1	69.4	60.6	81.6	72.9	64.9	37.0	2.7	34.0	15.6	29.3	7.5	12.2	11.6	15.3
SD	21.0	28.9	24.5	21.8	31.1	18.2	24.9	7.1	29.3	24.6	27.7	15.6	29.4	19.9	26.6
p***	0.44	0.31	0.45	**0.01***	0.60	0.27	0.13	**0.03***	0.16	0.17	**0.03***	0.41	0.80	0.08*	0.14
**No systemic treatment lines N=95**															
0 or 1 line – MW SD	82.8	69.1	59.0	76.7	73.0	63.9	37.8	5.4	32.1	16.1	36.1	8.8	10.8	14.9	17.5
SD	18.7	27.8	28.5	27.4	30.0	20.0	28.3	12.2	29.4	25.2	33.8	20.2	26.6	27.2	30.6
≥2 lines – MW SD	72.4	57.2	49.3	65.9	62.3	54.0	54.1	12.3	52.9	23.2	44.9	15.9	20.3	29.0	29.0
SD	26.3	34.4	30.1	33.5	33.8	21.3	30.0	22.0	32.4	34.0	34.2	24.3	31.4	29.0	40.6
p**	0.09*	0.09*	0.16	0.12	0.15	**0.04***	**0.02***	0.16	**<.01***	0.36	0.27	0.16	0.15	**0.03***	0.14
**Recurrence only surgery N =112**															
no	81.7	65.4	55.1	78.2	69.9	60.3	38.9	4.2	40.1	16.0	35.9	7.0	8.3	13.5	21.6
SD	18.1	29.5	29.4	29.1	32.1	23.2	29.4	10.4	33.4	24.2	35.5	19.1	22.7	24.0	33.9
yes	79.9	68.1	60.5	78.6	71.9	64.6	38.9	7.2	36.1	16.7	35.0	7.8	12.8	19.4	14.4
SD	23.8	30.9	25.5	25.1	30.9	20.6	30.0	16.1	31.9	27.8	32.7	15.5	25.4	26.2	29.4

p**	0.66	0.64	0.30	0.94	0.74	0.31	1.00	0.23	0.52	0.90	0.89	0.82	0.33	0.21	0.24
__Tumor location__															
Lower extremity incl hip/ pelvis	73.5	60.1	55.6	80.2	67.4	58.7	42.1	8.0	45.5	18.1	38.2	9.0	18.1	16.0	22.7
SD	26.3	35.0	26.3	24.5	34.0	23.8	30.3	18.2	34.2	27.5	33.7	15.0	29.9	26.6	31.9
Upper Extremity	76.0	54.2	57.6	72.2	58.3	62.5	43.5	2.8	44.4	13.9	47.2	8.3	22.2	5.6	33.3
SD	24.1	37.0	30.0	26.0	36.6	20.2	26.2	6.5	32.8	22.3	41.3	15.1	41.0	13.0	40.2
Trunk thoracic wall & back	83.2	64.8	59.6	75.9	69.9	63.5	37.4	1.9	38.3	27.2	45.7	11.1	7.4	13.6	15.4
SD	17.1	25.9	28.8	28.2	29.4	15.5	28.8	5.3	28.0	32.1	34.8	24.5	21.4	16.7	30.2
Abdominal wall	87.5	75.5	54.4	79.4	78.4	60.9	34.6	3.9	26.5	9.8	27.5	7.8	2.0	13.7	15.7
SD	11.5	25.8	34.8	35.1	32.7	19.2	29.6	9.4	27.0	15.7	29.4	25.1	8.1	20.6	35.6
Intra-abdominal	85.4	73.6	57.2	76.4	69.9	68.1	36.4	9.3	28.7	10.2	34.3	12.0	13.0	22.2	18.1
SD	18.8	28.0	28.1	23.4	29.5	20.1	31.3	15.7	30.8	20.8	34.3	21.3	26.8	33.8	30.6
Head & Neck	75.0	62.5	58.0	58.3	64.6	47.9	55.6	2.1	50.0	16.7	45.8	0.0	4.2	25.0	25.0
SD	18.8	21.4	25.2	35.6	28.8	28.1	26.6	5.9	32.1	35.6	35.4	0.0	11.8	34.5	38.8
not defined/ multifocal	87.6	69.0	53.6	73.8	69.1	61.9	44.4	2.4	35.7	23.8	38.1	4.8	4.8	14.3	19.0
SD	14.1	35.3	24.5	40.7	22.4	13.5	28.7	6.3	41.3	25.2	40.5	12.6	12.6	17.8	26.2
p***	_0.08_*	0.27	1.00	0.57	0.78	0.24	0.67	0.26	0.15	0.20	0.60	0.77	0.18	0.50	0.75
__Received treatments N=84__															
Surgery and/ or ST	84.2	70.1	59.8	77.0	73.1	63.9	37.7	5.6	32.3	15.9	34.9	9.5	13.2	15.9	19.4
SD	17.0	27.6	29.3	29.7	27.7	19.9	30.0	13.7	29.2	25.3	34.1	20.2	29.0	25.3	32.8
ST + RT + surgery	66.9	54.0	49.9	71.4	61.1	54.0	52.9	11.1	50.8	23.8	42.9	9.5	17.5	22.2	27.0
SD	29.5	35.7	30.6	29.4	38.1	21.8	25.3	20.0	32.3	33.6	33.6	18.7	32.7	33.9	38.9
p**	__0.02__*	__0.03__*	0.20	0.46	0.19	_0.06_*	__0.04__*	0.16	__0.02__*	0.26	0.36	1.00	0.58	0.36	0.38
__Education__															
basic/ medium	76.1	64.1	56.5	72.4	64.7	59.5	41.6	6.0	43.1	15.5	47.1	12.6	6.9	19.0	27.5
SD	24.0	31.2	26.5	27.0	32.8	22.6	29.0	13.5	31.8	25.1	35.3	21.5	20.5	27.3	36.3
high	82.8	66.5	56.7	78.8	71.8	63.2	39.5	5.7	35.2	17.7	33.3	7.3	15.3	14.9	15.6
SD	19.2	30.6	28.9	28.0	30.3	20.0	29.9	13.9	32.1	26.9	33.2	17.6	29.0	25.1	28.8
p**	_0.08_	0.64	0.96	0.17	0.17	0.30	0.67	0.89	0.14	0.62	__0.02__*	0.11	__0.04__*	0.35	__0.04__*
__Status Employment__															
employed	83.9	70.2	58.5	80.7	73.6	64.6	36.6	4.5	34.9	13.7	33.0	7.3	12.3	15.5	14.0
SD	17.8	27.6	26.6	25.7	27.7	19.0	27.8	12.0	29.3	23.8	30.9	17.0	26.3	24.4	27.1
unemployed, disability pension	54.4	36.3	37.7	43.1	33.3	39.1	71.9	15.7	67.7	29.4	72.5	21.6	19.6	27.4	62.5
SD	26.7	31.3	29.2	27.7	31.2	21.9	23.3	22.4	32.5	33.1	35.8	28.7	31.3	33.8	36.3
retirement pension	78.7	61.1	63.3	80.0	66.7	64.4	34.8	3.3	31.1	26.7	40.0	11.1	8.9	13.3	24.4
SD	20.7	34.3	28.7	21.1	36.2	21.5	29.4	9.3	35.6	31.4	33.8	20.6	26.6	30.3	32.0
other	87.4	74.1	58.3	79.6	79.6	63.9	32.1	7.4	31.5	14.8	40.7	7.4	3.7	11.1	18.5

SD	20.1	35.5	30.6	26.1	26.1	20.0	28.6	12.1	35.8	24.2	43.4	14.7	11.1	16.7	33.8
p***	**<.001***	**<.001***	**0.02***	**<.001***	**<.001***	**<.001***	**<.001***	**0.01***	**<.001***	*0.05**	**<.001***	**0.04***	0.47	0.28	**<.001***
